# FGF23 is elevated in multiple myeloma and increases heparanase expression by tumor cells

**DOI:** 10.18632/oncotarget.3794

**Published:** 2015-04-12

**Authors:** Attaya Suvannasankha, Douglas R. Tompkins, Daniel F. Edwards, Katarina V. Petyaykina, Colin D. Crean, Pierrick G. Fournier, Jamie M. Parker, George E. Sandusky, Shoji Ichikawa, Erik A. Imel, John M. Chirgwin

**Affiliations:** ^1^ Department of Medicine, Indiana University School of Medicine, Indianapolis, IN, USA; ^2^ Richard L. Roudebush VA Medical Center, Indianapolis, IN, USA; ^3^ Department of Pathology, Indiana University School of Medicine, Indianapolis, IN, USA

**Keywords:** multiple myeloma, osteocytes, FGF23, FGF receptor, klotho

## Abstract

Multiply myeloma (MM) grows in and destroys bone, where osteocytes secrete FGF23, a hormone which affects phosphate homeostasis and aging. We report that multiple myeloma (MM) cells express receptors for and respond to FGF23. FGF23 increased mRNA for EGR1 and its target heparanase, a pro-osteolytic factor in MM. FGF23 signals through a complex of klotho and a classical FGF receptor (FGFR); both were expressed by MM cell lines and patient samples. Bone marrow plasma cells from 42 MM patients stained positively for klotho, while plasma cells from 8 patients with monoclonal gammopathy of undetermined significance (MGUS) and 6 controls were negative. Intact, active FGF23 was increased 2.9X in sera of MM patients compared to controls. FGF23 was not expressed by human MM cells, but co-culture with mouse bone increased its mRNA. The FGFR inhibitor NVP-BGJ398 blocked the heparanase response to FGF23. NVP-BGJ398 did not inhibit 8226 growth *in vitro* but significantly suppressed growth in bone and induction of the osteoclast regulator RANK ligand, while decreasing heparanase mRNA. The bone microenvironment provides resistance to some anti-tumor drugs but increased the activity of NVP-BGJ398 against 8226 cells. The FGF23/klotho/heparanase signaling axis may offer targets for treatment of MM in bone.

## INTRODUCTION

Multiple myeloma (MM) is a hematological malignancy in which osteolytic bone disease due to tumor stimulation of osteoclasts and suppression of osteoblasts causes severe morbidity and is associated with poor prognosis [1]. Palliative treatments for MM bone disease rely on inhibitors of osteolysis such as bisphosphonates or RANK ligand-neutralizing antibody. Crosstalk between MM cells and bone remains incompletely understood, and direct molecular interactions between myeloma and factors unique to the bone microenvironment have not been described.

Osteocytes are the most abundant cell type within bone and source of the endocrine hormone FGF23. Unlike most fibroblast growth factors (FGFs), FGF23 lacks high affinity for heparin sulfate, which recruits ligands to FGF receptors (FGFRs). Assembly of FGFRs 1, 3 or 4 with transmembrane klotho forms specific receptors for FGF23 [2]. FGF23 acts on FGFR/klotho-expressing kidney tubule cells, where it regulates phosphate homeostasis and maintains the concentration of 1,25-dihydroxyvitamin D in its normal physiological range. Loss of FGF23 signaling may account for much of the aging phenotype of klotho-deficient mice, consequent to hyperphosphatemia and hypervitaminosis D, which can be ameliorated by dietary or genetic manipulations [3].

Klotho was discovered when an accidental gene inactivation in mice caused premature aging [4]. Klotho is an evolutionarily conserved protein with membrane-anchored and secreted forms and two major duplicated glycosyl hydrolase domains. In addition to serving as an FGF specificity co-receptor, klotho can inhibit insulin/IGF-1, Wnt and TGFβ signaling, suppress oxidative stress, and alter the activity of the TRPV5 calcium channel [5-8]. Over 80 publications have studied klotho in a variety of cancer types, with actions including effects on aging, inhibition of IGF-1, FGF2 and TGFβ signaling and tumor suppressor function [9, 10]. Tumor cells were not tested in these papers for the major physiological function of klotho to mediate cellular responses to FGF23.

FGFR signaling regulates the growth and progression of many cancers including MM [9], but a role for FGF23 in MM has not been reported. Since FGF23 is secreted by osteocytes, high concentrations of the factor will occur in the bone microenvironment - leading us to test a role for FGF23 in MM bone disease by asking if MM cells express receptors for and respond to FGF23.

## RESULTS

### FGF23 increases EGR1 and heparanase

**Figure 1:**
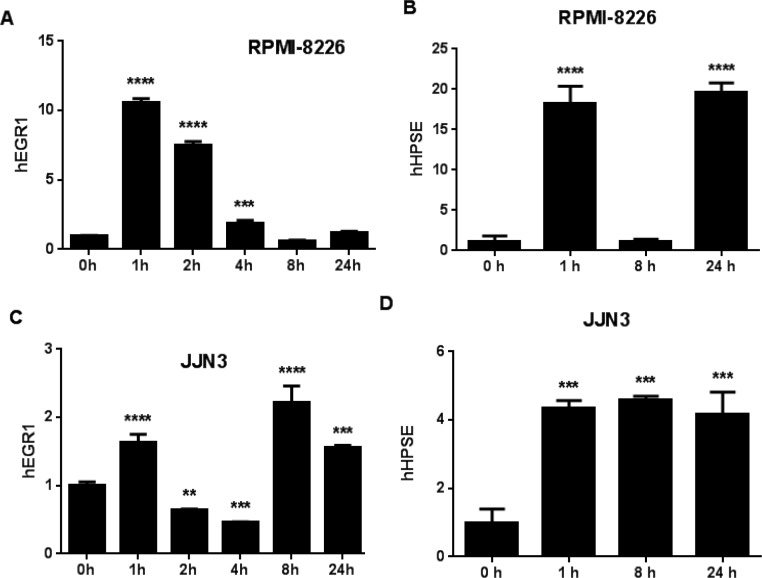
FGF23 regulates MM gene expression (**A**) and (**C**) FGF23 induces EGR1 in MM cells. Time courses (0-24hrs) of induction of EGR1 mRNA determined by PCR in RPMI-8226 (**A**) and JJN3 (**C**) myeloma cells in response to 100ng/ml FGF23. For both cell lines the increase in EGR1 mRNA at one hour was significant versus zero hours at *p* < 0.001. (**B**) and (**D**) Effects of FGF23 on heparanase (HPSE) expression. Time courses as in panels (**A**) and (**C**). Results for RPMI-8226 (**B**) and JJN3 (**D**) cells. Increases at 1 and 24 hours in heparanase mRNA were significant in both cell lines at *p* < 0.001.

Bone-metastatic breast and other cancer cell lines often express klotho [9], but tumor responses to FGF23 have not been reported. We asked whether MM cell lines responded to FGF23. FGF23 did not affect growth of MM cells *in vitro* (Supplementary Figure S1). In kidney tubules, FGF23 increases the early gene response transcription factor EGR1 [11]. When MM cell lines were treated with 100ng/ml FGF23, EGR1 mRNA was increased 2-10X at 1 hour and declined by 4 hours in RPMI-8226 and JJN3 (Figure 1A and 1C) and three additional MM cell lines (Supplementary Figure S2). A literature search for EGR1-responsive genes with roles in cancer and bone identified heparanase [12], an enzyme that significantly contributes to myeloma bone disease [13]. Heparanase mRNA was increased in RPMI-8226 and JJN3 cells 18-fold and 4-fold respectively by FGF23 (Figure 1B and 1D). We focused on these two human MM cell lines, since they cause osteolytic bone destruction in mouse models [14, 15]. Heparanase mRNA was unchanged in threeher MM cell lines (Supplementary Figure S2). The t5 alternate form of heparanase found in renal cancers [16] was not increased by FGF23 (Supplementary Figure S3).

### MM cells express klotho

**Figure 2:**
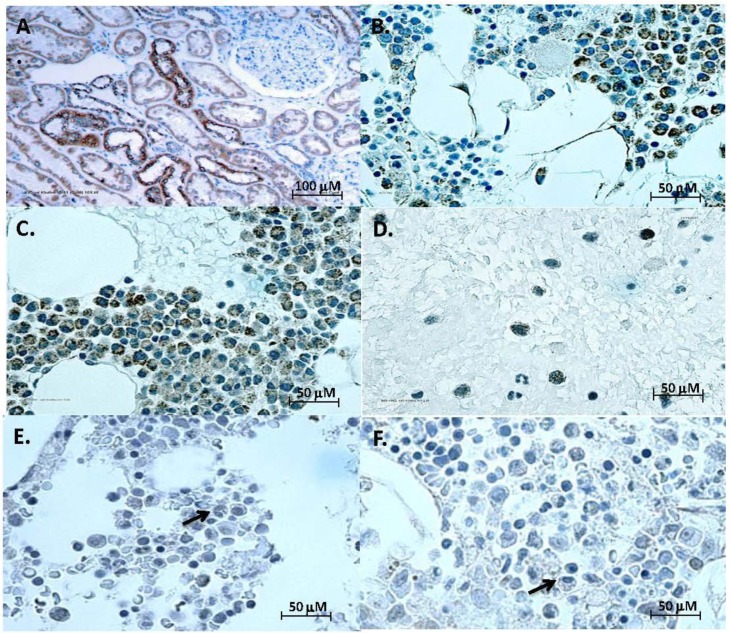

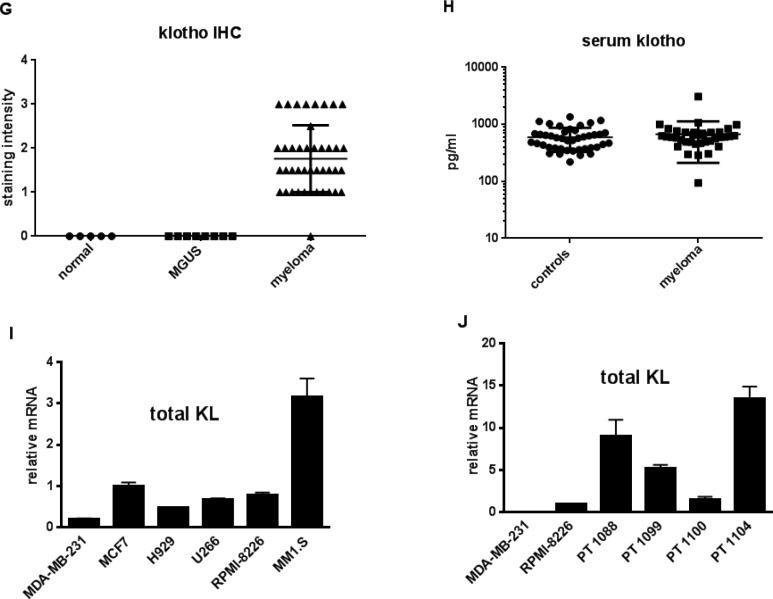
Klotho expression by multiple myeloma (**A**)-(**F**) Representative color images of klotho IHC. Kidney positive control (**A**), representative cases of multiple myeloma (**B**) and (**C**), bone marrow smear from a myeloma case (**D**), MGUS (E), and normal plasma cell (**F**), with arrows pointing at plasma cells. (**G**)-(**J**) Klotho staining and mRNA expression by MM. (**G**) Intensity of klotho IHC staining of plasma cells from normal (•) MGUS (▪) and myeloma (Δ) bone marrow samples (from Table 1) (*p* < 0.01 for MM *versus* normal or MGUS groups). Staining intensities were compared using ANOVA on ranks. (**H**) Serum concentrations of soluble klotho did not differ between 33 patients with MM and 43 controls (p=not significant). Y axis shows soluble klotho concentrations (pg/ml). Black horizontal lines represent mean klotho levels in each group. Vertical brackets represent ± one standard deviation. The distribution of variables was examined, and appropriate normalizing transformations were performed where needed for soluble klotho and serum intact FGF23 in the next Figure. Serum FGF23 and klotho concentrations in MM and controls were compared using unpaired t-tests for means. In addition, correlations between klotho or FGF23 levels and creatinine levels, Cockroft-Gault estimated glomerular filtration rate (GFR), as well as serum phosphate levels, were evaluated using Spearman correlation. (**I**) and (**J**) Klotho mRNA in MM. Real-time PCR for total klotho in four human MM cell lines (NCI-H929, U266, RPMI-8226, MM.1S) and breast cancer cell lines MDA-MB-231 and MCF7 [10]. Data are reported as fold differences in mRNA levels compared to that of breast cancer cells MCF-7, which express moderate klotho and are arbitrarily set at 1 (**I**). Klotho mRNA in 4 primary patient samples (PT1088, PT1099, PT1100 and PT1104) compared to that of 8226 MM cells set at 1 (**J**).

FGF23 signals by high affinity binding to complexes between a classical FGFR and klotho [2]. FGFRs are abundantly expressed in MM [17], but klotho has not been reported in myeloma cells, which we next tested. Bone marrow clots and aspirate smears from 42 patients with MM, 8 subjects with MGUS and 6 normal controls were stained with klotho antibody. Normal kidney was the positive control, with distal convoluted tubules staining intensely (Figure 2A). Klotho immunostaining was seen in plasma cells in all myeloma cases (Figure 2B and 2C). Klotho was localized to the cytoplasm of MM (Figure 2D) as punctate granules. In MGUS, there was minimal to no cytoplasmic staining in occasional plasma cells (Figure 2E) and no staining of plasma cells in normal bone marrow (Figure 2F). Compared to non-MM plasma cells, klotho expression by MM cells was significantly increased (*p* < 0.01, Figure 2G) when staining was scored blind on a standard scale. No relationship was observed between percent MM cells in bone marrow and intensity of klotho staining. No significant association was observed between the klotho staining and disease features, including staging and extent of bone involvement.

### Serum klotho is unchanged in MM

We asked if serum soluble klotho was altered in MM, using an ELISA that recognizes secreted and shed forms [18]. Detection of both forms was confirmed with supernatants from cultures of breast cancer cells transfected with membrane-bound, secreted (549 amino acid) or the 980 amino acid extracellular domain of klotho (Supplementary Information). High concentrations of klotho were found in similar amounts in media from cells expressing each of the forms of klotho (data not shown). Soluble klotho concentrations did not differ (*p* = 0.39) between MM patients (*n* = 33, mean ± SD = 670 ± 458 pg/ml) and controls (*n* = 43, 598 ± 269 pg/ml) (Figure 2H). Concentrations of soluble klotho were below the limit of detection (<6.15 pg/ml) in media conditioned by four MM cell lines (data not shown). We found klotho mRNA in four human MM cell lines (Figure 2I) and purified CD138^+^ primary cells from four patients (Figure 2J). The cell lines expressed both membrane and secreted klotho mRNAs (Supplementary Figure S4). Patient samples and cell lines also expressed mRNAs encoding at least two of the three FGFRs that couple to klotho (Supplementary Figure S5).

### Serum intact FGF23 is increased in MM

**Figure 3:**
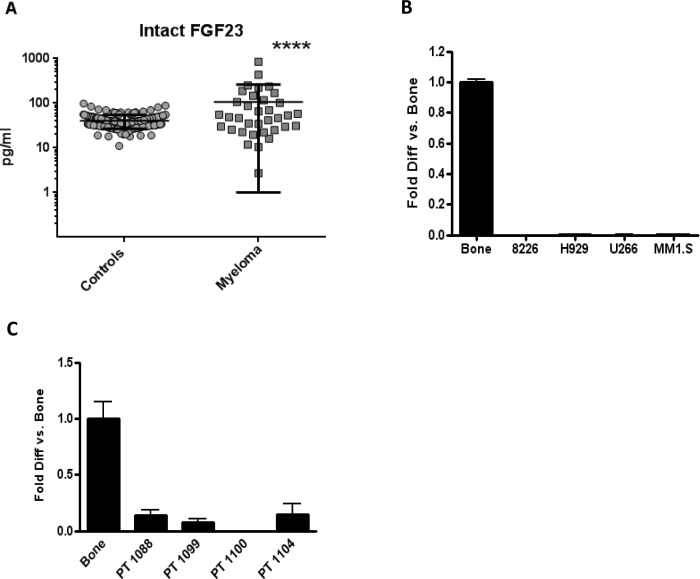
Serum intact FGF23 was higher in MM patients compared to controls, while MM cells made little FGF23 mRNA (**A**) Serum intact FGF23 concentrations (pg/ml) from 186 controls and 33 MM patients, determined by ELISA. Mean concentrations were 2.9 fold higher in patients with MM (*p* < 0.0001) compared to controls. Vertical brackets indicate ± 1 standard deviation from the mean (black horizontal lines). FGF23 mRNA levels in MM cell lines (**B**) and four primary patient samples (**C**) by real-time PCR. Human bone total RNA was the positive control for FGF23.

Mean Intact FGF23 concentrations were 2.9 fold higher in 33 newly diagnosed MM patients compared to 186 controls (106 ± 157 pg/ml and 36 ± 17 pg/ml; *p* < 0.0001) (Figure 3A). MM is a relatively rare malignancy: our patient population was sufficient to provide statistical significance, but not large enough to show differences between subgroups when the MM samples were stratified according to degree of bone involvement (Supplementary Figure S6). To test if myeloma cells were a source of circulating FGF23, cell lines and primary MM cells were analyzed by real-time PCR. FGF23 messenger RNA was undetectable (four myeloma cell lines, Figure 3B) or much lower (four primary patient samples, Figure 3C) than the positive bone control.

### Co-culture with MM increases FGF23 expression in bone

**Figure 4:**
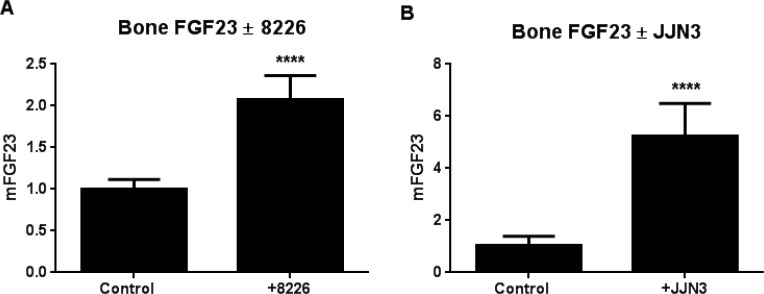
MM cells induce bone expression of FGF23 Myeloma cells (10^4^) were co-cultured with calvarial bone sections from B6.SJL-*Ptprc^a^Pep3^b^*/BoyJ mouse pups for 7 days and expression of bone FGF23 analyzed by Q-PCR with species-specific primers for mouse FGF23. Contact with RPMI-8226 (**A**) or JJN3 (**B**) human MM cells significantly increased bone FGF23 mRNA (*p* < 0.001).

The co-culture assay modifies a standard procedure for growing mouse bone *ex vivo* [19], by the addition of human tumor cells [20]. We adapted it for MM cell lines and analyzed changes in tumor and bone markers after one week by species-specific real-time PCR to distinguish between mouse bone and human MM mRNAs. RPMI-8226 or JJN3 human MM cells significantly increased mouse FGF23 mRNA 2-fold and 5-fold respectively, compared to bones without added MM cells (Figure 4).

### FGFR inhibition affects tumor growth

**Figure 5:**
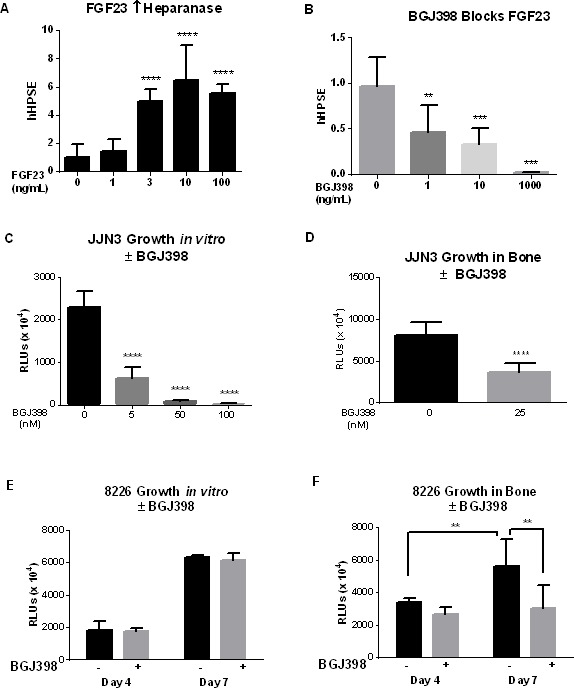
Pan-FGFR kinase inhibitor blocks responses to FGF23 *in vitro* and growth of MM in bone (**A**) Dose-response to FGF23 of RPMI-8226 cells for induction of heparanase mRNA at 24hrs. Cells treated with recombinant human FGF23 between 1 and 100ng/ml and analyzed as in Figure 1C. (**B**) Increased heparanase mRNA in RPMI-8226 cells in response to 10ng/ml FGF23 was dose-dependently blocked by the pan-FGFR kinase inhibitor NVP-BGJ398. (**C**)-(**E**) MM tumor burden assayed as secreted *Gaussia* luciferase from stably transduced cells. (**C**) Dose-dependent inhibition of JJN3 cells *in vitro* by NVP-BGJ398. (**D**) Inhibition by 25nM NVP-BGJ398 of 5,000 JJN3 cells added to Swiss Webster neonatal mouse calvariae. (**E**) Lack of inhibition of RPMI-8226 cells in tissue culture by 40nM NVP-BGJ398. (**F**) Inhibition at 4 and 7 days with 40nM NVP-BGJ398 of 5,000 8226 MM cells in bone, as in (**D**), with calvariae from B6.SJL-*Ptprc^a^Pep3^b^*/BoyJ mice: significant versus control at *p* < 0.01.

Next we tested if an inhibitor of FGF23 signaling altered myeloma growth in bone. The dose-response to FGF23 was first determined *in vitro*. FGF23 at 3ng/ml (1.2×10^−10^M) significantly induced heparanase mRNA (Figure 5A). The pan-selective FGFR inhibitor NVP-BGJ398 dose-dependently blocked the response to 10ng/ml FGF23 (Figure 5B), from which we chose 25 or 40nM (14 or 22ng/ml) to test against growth of MM cells stably secreting *Gaussia* luciferase [21] to follow tumor burden [22]. The drug dose-dependently inhibited growth of JJN3 cells *in vitro* at 7 days (Figure 5C), while 25nM decreased MM activity in bone over the same time (Figure 5D). In contrast, RPMI-8226 cells were resistant to 40nM inhibitor *in vitro* (Figure 5E), but the same concentration significantly decreased their activity in bone (Figure 5F).

### FGFR inhibition alters MM and bone gene expression

**Figure 6:**
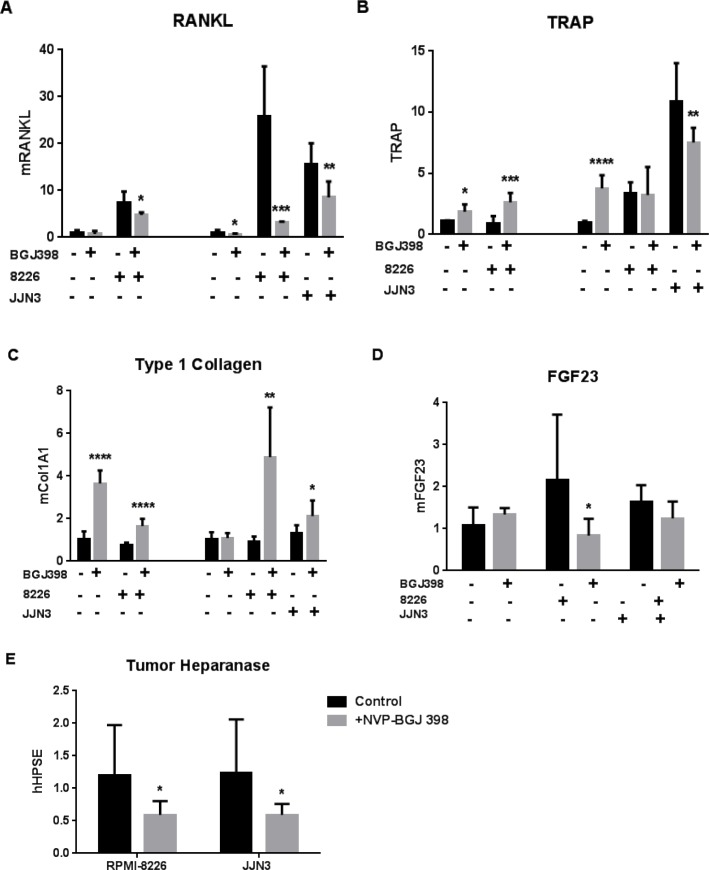
Effects of pan-FGFR kinase inhibitor on bone cell and MM responses in co-culture Species-specific PCR analyses of calvariae incubated for 7d with 5,000 RPMI-8226 or JJN3 MM cells and treated ± NVP-BGJ398. (**A**)-(**D**) Expression of bone genes analyzed with mouse-specific primers. (**A**) Regulator of osteoclastogenesis, RANK ligand. (**B**) Osteoclast marker, TRAP. (**C**) Osteoblast biosynthetic activity marker, type 1 collagen. (**D**) Osteocyte marker, FGF23. (**A**)-(**C**) Combine data from separate experiments with B6.SJL-*Ptprc*^a^*Pep3*^b^/BoyJ mouse calvariae (left-hand four bars with [NVP-BGJ398] = 40nM) and Swiss Webster calvariae (right-hand six bars, with [NVP-BGJ398] = 25nM). (**E**) Myeloma cell expression of human heparanase in presence of Swiss Webster calvariae ± 25nM NVP-BGJ398. Comparisons for significance are between untreated and treated pairs. (**E**) includes convention for bar labeling used in all panels.

Finally, we asked if FGF signaling inhibition altered bone responses to tumor. The co-cultures from Figure 5D and 5F were processed for RNA isolation and analyzed by Q-PCR with mouse-specific primers for bone markers: RANKL, the central regulator of osteoclastogenesis (Figure 6A) and tartrate-resistant acid phosphatase (TRAP) a major osteoclast enzyme (Figure 6B) were increased by myeloma cells. The increase in RANKL was opposed by NVP-BGJ398 treatment. TRAP was less affected, but osteoclast activity may have peaked before day 7 when the cultures were assayed. Type 1 collagen, a major product of metabolically active osteoblasts was increased by drug treatment in the presence of MM cells (Figure 6C), while NVP-BGJ398 had a modest effect to oppose the increase of FGF23 in the presence of MM cells (Figure 6D). Finally, we found that the pan-FGFR inhibitor decreased the FGF23 target heparanase in the two MM cell lines grown in bone, when analyzed by Q-PCR with human-specific primers (Figure 6E).

## DISCUSSION

Greater than 80% of patients with multiple myeloma develop osteolytic bone disease, due to osteoclast activation and osteoblast suppression, leading to skeletal-related events, including severe bone pain, vertebral compression and pathologic fractures [1]. No specific molecular actions on MM by factors *unique* to bone have been reported previously, prompting us to ask if such factors might contribute to myeloma growth in bone. We focused on osteocytes as the most abundant cell type in the skeleton and main source of bone-derived endocrine factors, including FGF23 [23].

To test if FGF23 activates signaling in myeloma cells, we assessed EGR1, an early response transcription factor induced by FGF23 in kidney tubules [11]. EGR1 mRNA was rapidly increased in five MM cell lines (Figure 1A and 1C and Supplementary Figure S2A, S2C and S2E), while growth or apoptosis *in vitro* of three human MM cell lines was unaffected by FGF23 treatment (Supplementary Figure S1 and data not shown). EGR1 transcriptionally regulates a plethora of genes in many cell types [24]. In prostate cancer it is tumorigenic and pro-metastatic, activating secretion of growth factors and enzymes that modify extracellular matrix. A significant transcriptional target of EGR1 is heparanase [12, 25], a central regulator of osteolysis and growth in bone of myeloma and solid tumors [13] (additional references in Supplementary Information). FGF23 strongly increased heparanase in RPMI-8226 and JJN3 MM cell lines (Figure 1B and 1D). The short, alternative t5 form of heparanase expressed by renal cancers [16] was not increased by FGF23 (Supplementary Figure S3). The results show that FGF23 has direct actions on MM cells. In prostate or breast adenocarcinoma and colon carcinoma cell lines de Mestre et al [12] observed that heparanase mRNA was increased at 24hrs, by which time EGR1 mRNA (stimulate by PMA treatment of the cells) had returned to baseline - as expected for an early response gene. In Figure 1 we observed the same temporal relationship between EGR1 and heparanase in FGF23-treated MM cell lines. The MM cell lines that did not show increased heparanase mRNA upon treatment (Supplementary Figure S2) expressed much higher basal message than the JJN3 and RPMI-8226 cell lines that showed strong induction of heparanase by FGF23.

Heparanase is a centrally important regulator of metastases, tumor microenvironment interactions, and bone metabolism via actions on many targets [25, 26]. High heparanase is an indicator of poor prognosis in MM [27]. It increases local expression of proteases [28] and RANK ligand [13], the central regulator of osteoclasts. It also suppresses osteoblast activity [29] - a hallmark of myeloma bone disease [1].

We asked if myeloma cells express FGF23 receptors [11]. FGF23 lacks high affinity for heparan sulfate proteoglycans, which increase FGFR affinity for conventional FGF ligands. Instead, a C-terminal extension of FGF23 confers high affinity for the klotho:FGFR complex [2]. Klotho is expressed in many tumor types, increasing with progression in ovarian cancer [30] while decreasing in breast cancer [10]. MM express FGFRs [17, 31], but klotho expression has not been reported. We found klotho IHC staining in all MM samples tested, while a small sample of normal plasma cells and MGUS bone marrow aspirates was negative (Figure 2B-2G) with a well-characterized monoclonal antibody [32] previously used in kidney (Figure 2A) and in small cell lung cancer samples [33]. While klotho is a transmembrane protein, its immunostaining was frequently intracellular (Figure 2D). The klotho:FGFR complex could be endocytosed upon ligand binding, causing the punctate cytoplasmic staining observed. Figure 2G suggests that klotho expression (and potential FGF23-responsiveness) may be a hallmark of the transformation of plasma cells into MM. Normal plasma cells from additional patients need to be analyzed to confirm this. FGF23 signaling ensues when ligand binds to klotho plus FGFRs 1, 3 or 4 [34]. Four standard human MM cell lines and four primary patient samples all expressed klotho mRNA (Figure 2I and 2J). CD138^+^ primary MM cells expressed FGFRs 3 and 4 but not significant amounts of FGFR1, while the cell lines RPMI-8226 and JJN3 expressed FGFRs 1 and 4 (Supplementary Figure S5), predicting that all should be responsive to FGF23.

Stewart et al previously reported that serum FGF23 levels were increased in patients with myeloma and correlated with serum paraprotein [35]. The authors' ELISA used polyclonal antibodies against C-terminal epitopes, consequently detecting both biologically active intact FGF23 and inactive C-terminal fragments [36]. Only intact FGF23 activates the membrane klotho:FGFR complex. C-terminal fragments may compete with intact FGF23 for receptor binding and have antagonist functions [37]. Imel et al demonstrated that in healthy subjects, C-terminal FGF23 concentrations are frequently elevated when intact FGF23 and phosphorus concentrations remain normal [38]. Thus C-terminal FGF23 and intact assays provide different information. We found increased intact FGF23 (Figure 3A) in MM serum but cannot exclude the presence of inactive FGF23 fragments. Stewart et al [35] proposed that MM cells make FGF23 on the basis of immunostaining, which could be due to binding of exogenous FGF23 to receptors or nonspecific binding. Ectopic FGF23 expression has only been reported in rare mesenchymal tumors associated with hypophosphatemic tumor-induced osteomalacia, due to renal phosphate loss [39] - a feature not seen in myeloma. We failed to detect significant amounts of FGF23 mRNA in MM cell lines or primary MM samples (Figure 3B and 3C). Our CD138-positive primary cells were rapidly purified by cell sorting from marrow aspirates to decrease bone cell contamination as a source of FGF23. It is not possible to distinguish myeloma cells being devoid of FGF23 mRNA but contaminated with bone cells, *versus* low expression of FGF23 by MM cells. A larger controlled study is required to resolve this issue. We conclude that MM cells are not a significant source of FGF23. We did not observe differences in FGF23 levels between groups with different degrees of bone involvement (Supplementary Figure S6). The relationship of serum FGF23 to extent of bone involvement needs to be tested in a larger cohort of MM patients using accurate assessment of bone involvement.

The presence of myeloma cells could stimulate osteocyte secretion of FGF23, leading to its elevation in patient sera. We tested if MM cells increased bone production of FGF23, using a novel *ex vivo* assay where tumor cells are added to primary bone organ cultures [19]. Neonatal mouse calvariae are immune-naïve and support growth of human solid tumor cell lines [20] that cause bone metastases in mouse models. We added small numbers of MM cells and monitoring gene changes in tumor cells (human) and bone cells (mouse) by real-time PCR with species-specific primers [40] to distinguish the cellular source of mRNAs in the mixed species co-cultures. Bone expression of FGF23 mRNA was significantly increased 2-5 fold after one week of co-culture with myeloma cells (Figure 4). Thus, increased circulating FGF23 in patients may result from MM stimulation of osteocytes. Studies are under way to identify myeloma-derived factors that increase FGF23 production. Other factors that might affect circulating FGF23 concentrations include bisphosphonates, renal function and iron status. However, patient bisphosphonate use decreases FGF23 [41]. Despite high intact FGF23 levels, serum phosphate concentrations were largely normal in our sample set, suggesting that other factors maintain phosphate homeostasis. Iron deficiency correlates with elevated C-terminal FGF23 [42]. Imel et al reported, in a study of autosomal dominant hypophosphatemic rickets (ADHR), that low serum iron was associated with elevated C-terminal but not intact FGF23 in control subjects, while increased intact molecule was also negatively correlated with iron in ADHR patients. The results suggest that FGF23 cleavage is a compensatory mechanism to maintain active FGF23 in the normal range [38]. MM-related anemia is due to impaired iron utilization, rather than low iron *per se* [43], with most MM patients having normal or high iron status, making it unlikely to explain high FGF23. Parathyroid hormone receptor activation increases bone expression of FGF23 [44], and MM cells may secrete its ligand, PTHrP [45]. Factors that can influence FGF23 levels should be explored in a larger cohort of MM patients, including 1,25-dihydroxyvitamin D, PTHrP, other FGFs, low pH and high serum phosphate/calcium ratio [46].

To test a role for FGF23 in myeloma bone disease, we used the pan-FGFR tyrosine kinase inhibitor NVP-BGJ398, which rapidly normalized phosphate and enhanced bone growth - with increased mineralization and normalization of growth plate structure - in a mouse model of ADHR, where elevated FGF23 causes hypophosphatemia [47]. NVP-BGJ398 effectively blocked the heparanase response of MM cells *in vitro* to 10ng/ml FGF23 (Figure 5B). JJN3 MM cells were highly sensitive to growth inhibition *in vitro* by NVP-BGJ398 and were also growth inhibited in bone co-culture, while 40nM NVP-BGJ398 had no effect on the growth of RPMI-8226 cells in standard tissue culture but blocked their growth in bone, as monitored by secreted luciferase marker (Figure 5E *versus* 5F). The bone microenvironment frequently confers resistance to treatment; therefore the increased sensitivity of 8226 to NVP-BGJ398 in bone compared to *in vitro* is of particular interest. It suggests that FGF targeting may alter the bone microenvironment, making it hostile to tumor growth - although NVP-BGJ398 showed few effects on bone alone. In MM:bone cocultures, it decreased tumor RANKL, increased osteoblastic activity (type 1 collagen) and decreased osteocyte FGF23 - which in turn should decrease tumor heparanase. Additional work is needed to determine if NVP-BGJ398 acts primarily through direct blockade of FGF23 signaling or indirectly through other actions on tumor, bone, or both.

NVP-BGJ398 and several other pan-FGFR kinase inhibitors are in clinical development for cancer treatment [31, 48]. Inhibition of FGFR signaling in MM has focused on cells carrying activating FGFR3 mutations that cause ‘addiction' to FGFR signaling and confer sensitivity to FGFR inhibitors. A recent clinical trial of Dovitinib, a multikinase inhibitor with activity against FLT3/c-Kit, FGFR1/3 and VEGFR1-4 kinases, in patients with relapsed or refractory MM showed marginal efficacy in subjects with or without FGFR3 mutations [49]. The reason for the treatment failure is unclear, and correlative data on FGFR inhibition were not reported. Participating patients were heavily pretreated and some died quickly due to disease progression; so the results do not exclude FGFRs as viable targets for MM treatment. We chose NVP-BGJ398 on the basis of its ability to block FGF23 in an animal model, where it caused anabolic bone responses [47] - which could contribute to the antitumor effects seen in bone. NVP-BGJ398 and other tyrosine kinase inhibitors selective for FGFRs, including LY2874455, SSR128129E, AZD4547 and PD173074, are in early clinical development for solid tumors. FGF signaling is complex and variable in different tumors at different stage. Better understanding of FGF signaling in MM and bone should clarify the potential of FGFR inhibitors for MM treatment.

Our results suggest that FGF23 from skeletal osteocytes drives a vicious cycle [50] of myeloma growth in bone by binding to MM cells and activating transcription of pro-metastatic and pro-osteolytic genes, such as heparanase. Future research is needed to identify additional genes regulated by FGF23 and to clarify the mechanisms by which MM systemically increases active FGF23 in patients. The action of FGF23 on MM cells identifies targets for intervention against myeloma bone disease, including heparanase antagonists [51, 52], FGF23-neutralizing antibodies [53] and FGFR kinase inhibitors [48], now entering clinical trials.

## MATERIALS AND METHODS

### Cell lines and materials

Human MM cell lines RPMI-8226, U266, NCI-H929 and MM.1s and human breast cancer cell lines MCF-7 and MDA-MB-231 were from the ATCC and grown in recommended media. JJN3 human MM cells were from Professor N. Giuliani, University of Parma. Recombinant human FGF23 was from R&D Systems Inc. Anti-klotho antibody KM2076 is a rat IgG(2)a monoclonal antibody raised against residues 55-261 of human klotho protein [32] and was generously provided by Kyowa Hakko Kirin Co. Ltd., Tokyo; it is now available commercially. NVP-BGJ398 was from ChemieTek, Indianapolis, IN.

### Human Specimens

All patient materials were used with informed consent and HIPAA compliance with a protocol approved by the Indiana University Institutional Review Board. Previously frozen serum samples obtained at diagnosis were available from 33 MM patients (Table 1). Serum FGF23 and soluble klotho data for healthy controls were previously analyzed by one of the authors (EAI) and used for comparison with myeloma samples. Serum klotho results were available from 43 and FGF23 from 186 control subjects [38]. Klotho in bone marrow plasma cells was evaluated in paraffin-embedded bone marrow clots, bone marrow aspirate smears and tonsil tissues from the histology archives of the Department of Pathology. Bone marrow specimens from 42 newly diagnosed MM patients, 8 MGUS patients, 6 healthy controls, and two normal tonsils were stained for klotho immunohistochemistry (IHC). Of these 42 patients, 15 had corresponding serum samples at the time of diagnosis available for klotho and FGF23 analyses. Patient clinical parameters are summarized in Table 1.

**Table 1:** Patient characteristics

Bone marrow samples			
Characteristics	Newly diagnosed Myeloma (n=42)	MGUS (n= 8)	Normal (n= 6)
Age (years)	41-76 (median = 63)	56-68 (median 59)	45-67 (median 56)
Sex (Male/Female)	25/17	5/3	3/3
Stage (percent) I II III	16 (38%) 16 (38%) 10 (24%)	N/A	N/A
Bone involvement (percent) No lytic lesions <5 lesions ≥ 5 lesions	8 (19%) 7 (17%) 27 (64%)		
**Serum Samples**	**Newly diagnosed MM (n=33)**		
Age	41-78 (median 62)		
Sex (Male/Female)	20/13		
Stage (percent) I II III	16 (48%) 11 (33%) 6 (18%)		
Bone involvement (percent) No lytic lesions <5 lesions ≥ 5 lesions	7 (21%) 6 (18%) 20 (60%)		
Serum creatinine (mg/dl)	0.55-2.50 (Mean=1.20 ± 0.5)		
Cockroft-Gault estimated glomerular filtration rate (ml/minute)	25 to 102 (Median = 53)		
Serum phosphate (mg/dl) (n=25)	1.80-5.83 (mean 4.2 ± 1.0)		

No statistically significant association was observed between the klotho staining and disease features, including staging and extent of bone involvement. In our sample set where most patients have normal or mild renal insufficiency (based on serum creatinine and Cockroft-Gault Estimated GFR), we did not observed a correlation between serum intact FGF23 and serum klotho, serum creatinine, Cockcroft-Gault calculated GFR, or serum phosphate levels (p>0.05). In 15 cases where both serum and bone marrow samples were available, there was no correlation between serum klotho concentrations and klotho expression by immunostaining.

### Serum assays

Intact FGF23 was assayed as described [54] using ELISA kits from Kainos Laboratories (Tokyo, Japan) with a lower limit of detection of 3pg/ml and coefficient of variation of 6%. The assay recognizes only intact FGF23 via monoclonal antibodies for epitopes on opposite sides of the FGF23 R^176^XXR^179^ cleavage site. Soluble klotho in sera and conditioned media was assayed using an ELISA (IBL-America), with a lower limit of detection of 6.15pg/ml and coefficient of variation of 5% [18]. Creatinine, calcium and alkaline phosphatase were measured in the clinical pathology laboratory using standard methods. Glomerular filtration rates were calculated using the Cockroft-Gault equation. Serum phosphate was measured with a Roche COBAS Mira S (Roche Diagnostics), using reagents from Thermo Scientific. Serum phosphate values >6ng/dl were presumed to be secondary to either hemolysis or interference with the analytical reading by excess monoclonal protein and were excluded. 25 MM subjects had assessable serum phosphates.

### Immunohistochemistry

Procedures used solutions, instruments and protocols from Dako. Sections were cut and placed on positively-charged slides. After removal of paraffin and washing with distilled water, the slides underwent antigen retrieval using Target Retrieval high pH solution for 20 min at 90°C and blocking for 20 min with serum-free protein block solution before adding anti-klotho antibody 2076 (1:500) for 20 min. The secondary antibody was donkey anti-rat IgG conjugated to horseradish peroxidase (1:10, Jackson ImmunoResearch Labs) for 10 min, followed by 10 min incubation with EnVision™ FLEX+ high-sensitivity visualization system in an Autostainer. Five min after addition of 3,3′-diaminobenzidine chromogenic reagent, the slides were counterstained with hematoxylin and examined under a Leica bright field microscope. Five microscopic fields with the highest immunoreactivity were evaluated independently by three investigators in blinded fashion. At least 1,000 cells were analyzed per slide. The number of positively-staining cells was estimated, and the intensity of staining was scored on a scale of 0 to 3+ (0, no staining; 1, weak staining; 2, moderate staining; and 3, strong staining). Kidney was the positive control.

### RNA and PCR

Tumor cells (10^4^/well) were grown in RPMI1640 medium and treated with FGF23, collected by centrifugation and washed in phosphate-buffered saline (PBS). Plasma cells were isolated from bone marrow aspirates using magnetic beads that bind CD138 (syndecan-1)-positive cells, according to the manufacturer's instructions (Miltenyi Biotech), yielding > 90% pure CD138^+^ cells by flow cytometry. RNAs were extracted from cells from four patients and from MM cell lines using Qiagen RNeasy mini kits with DNAse treatment, converted to cDNA using Omniscript RT kits (Qiagen) with 16mer oligo(dT) primer and analyzed in triplicate by real-time (Q-)PCR using Qiagen Quantitect SYBR green PCR kits and a Biorad iCycler single-color real-time detection system. Ribosomal protein L32 was the normalization control [40]. The sequences, design strategy and validation for PCR primer sets are given in the Supplementary Information. For species-specific PCRs, primer sets were tested against mouse and human positive templates until sets that give 1000:1 specificity were found [40]. Relative mRNA expression was calculated using the ΔΔCT method [55].

### Co-culture of MM cells with bone

MM cell lines were transduced with lentiviral particles from GenTarget, according to the manufacturer's instructions, with a vector encoding secreted *Gaussia* luciferase driven by a CMV promoter, plus a GFP-blasticidin fusion protein cassette. GFP^+^ MM cells were isolated by fluorescence-activated cell sorting. Segments of neonatal mouse bone were prepared by a modification of Mohammad et al [19]. Calvariae from euthanized 11 to 14 day-old mouse pups were cut into 4mm disks with a biopsy punch and placed into uncoated 24-well plates. Luciferase-secreting GFP-MM cells (1×10^4^) were added in 0.5ml BGJb medium (Life Technologies) supplemented with 20% fetal calf serum, 500nM human insulin and 100μM ascorbic acid. Plates were incubated at 37°C in 5% CO_2_. Medium was changed every 2-3 days and assayed for luciferase as an indicator of tumor burden [21, 22] with a BioLux® *Gaussia* luciferase flex kit (NE BioLabs) and a Turner TD 20/20 luminometer. Results were expressed as relative luminescence units (RLUs). At day 7, bones were washed in PBS, placed in 1.0ml of Qiazol (Qiagen) and homogenized for 1 min with zirconium beads in a BeadBug microtube homogenizer (Benchmark Scientific) at 280 strokes/min, followed by RNA isolation.

### Statistics

Analyses were performed using Graphpad Prism 5 and 6. Experiments were performed in replicate and results reported as means ± standard deviation, where applicable. Expression of mRNA was compared between groups using one-way ANOVA with Tukey's multiple comparison post-test. Results are shown with mean ± standard deviation, where applicable. Degree of significance was represented using *p* values (* = *p* < 0.05, ** = *p* < 0.01, *** = *p* < 0.001, **** = *p* < 0.0001).

## SUPPLEMENTARY FIGURES



## Abbreviations

ADHR: autosomal dominant hypophosphatemic rickets
EGR1: early gene response (transcription factor) 1
FGF: fibroblast growth factor
FGFR: FGF receptor
FLT3: Fms-related tyrosine kinase 3
IHC: immunohistochemistry
MGUS: monoclonal gammopathy of undetermined significance
MM: multiple myeloma
PTHrP: parathyroid hormone-related protein
Q-PRC: real-time PCR
RANK: receptor activator of nuclear factor kappaB
RANKL: RANK ligand
TRAP: tartrate-resistant acid phosphatase
