# Supplementary material for: FGF23 is elevated in multiple myeloma and increases heparanase expression by tumor cells
Source: Oncotarget. 2015 Apr 12;6(23):19647–60. doi: 10.18632/oncotarget.3794 (PMC4637311; doi:10.18632/oncotarget.3794)
Supplement: Supplementary file 1 [file oncotarget-06-19647-s001.pdf]

FGF23 is elevated in multiple myeloma and increases heparanase expression by tumor cells

Supplementary Material

The MM cell lines were obtained over ten years ago, at which time they were authenticated by karyotyping at the ATCC. Upon receipt, the cell lines were expanded and stored as primary stocks in liquid N_2. Cells from primary stocks were grown for no longer than 6 months with semi-monthly testing for mycoplasma. Subclones (such as luciferase-secreting stable transductants) were similarly stored as primary stocks and grown for no longer than 6 months. Human klotho was expressed from cDNAs subcloned into pcDNA3.1/V5/His-TOPO (Invitrogen) to generate fusion proteins with C-terminal epitope extensions. Expression plasmids encoding membrane (1012 amino acid, Genbank AB005142) and soluble (549 amino acid, Genbank AB009667) isoforms were provided by Dr HE Dietz, Johns Hopkins University. A variant lacking the C-terminal transmembrane domain (980 amino acids + C-terminal extension) was generated by mutagenesis *in vitro.* A QuickChange site-directed mutagenesis kit was used according to manufacturer's instructions (Stratagene).

Species-specific PCR primers were designed with online Primer 3 tool (**http://bioinfo.ut.ee/primer3/**) and tested for species-specificity with the NCBI primer design tool (http://www.ncbi.nlm.nih.gov/tools/primer-blast/) by Blast searching against the targeted sequence and versus both *Mus musculus* and *Homo sapiens* sequence databases to eliminate cross-species and erroneous amplifications, and allowing amplification of transcript variants. The templates used for primer design were Genbank RefSeq files for the designated genes. The NCBI primer design tools proved unsatisfactory for the initial design of primers suitable for species-specific amplification. Primers were then separately tested using real-time PCR with cDNAs from mouse calvariae and human tumor cells. Primer sets were accepted only when they yielded results on the proper species, gave single melt curve peaks and a cycle efficiency of >95%, Ct values of less than 33 and only produced one band of DNA of the appropriate size by gel electrophoresis. All PCR data were normalized to the house-keeping controls RPL32 specific for mouse or human. Lower case m or h in front of gene symbol indicates mouse or human specificity. Human heparanase is encoded by HPSE and file used was Accession NM_006665 encoding transcript variant 1. Human EGR1 used Accession: NM_001964. Human RPL32 used Accession NM_000994 for transcript variant 1. Human FGF23 used Accession NM_020638. Human klotho used Accession NM_004795. Mouse RPL32 used Accession NM_172086. Mouse FGF23 used Accession NM_022657. Mouse RANKL (Tnfsf11) used Accession BC131970.1. Mouse TRAP (Acp5) used Accession BC019160.1. Mouse type 1 collagen alpha1 (Col1a1) used Accession NM_007742.3.

hRPL32: forward = 5'-TCAAGGAGCTGGAAGTGCTG; reverse = 5'-TGCACATGAGCTGCCTACTC

total hKlotho: forward = 5'-CCTCATGGATGGTTTCGAGT; reverse = 5'- GGAGGGAAGCCATTTTTCTC

membrane hKlotho (1012 amino acid splice form): forward = 5'-GTGGCACAGAGGTTACAGCA;
reverse = 5'-GTGGTATCTACTTGAATGTA

secreted hKlotho (549 amino acid splice form): forward = 5'-GTGGCACAGAGGTTACAGCA;
reverse = 5'-GGTGAGACTGCTGATTGGTT

hEGR1: forward = 5'-GCCATAGGAGAGGAGGGTTC; reverse = 5'-GTTGGCCAATAGACCTTCCA

hFGF23: forward = 5'-TGCCCTGCTAGAATTTGCTT; reverse = 5'-TTCTTTCCCCCTGAGTCCTT

hHPSE: forward = 5’-CCCTCCTGATGTGGAGGAGA; reverse = 5’-TGGTAGCAGTCCGTCCATTC

hT5-HPSE: forward =5’- TGGCACCAAGACAGACTTCC; reverse = 5’- GCCCGAGTCCAACCTATTCA

mRPL32: forward = 5’-GCTGCCATCTGTTTTACGGC; reverse = 5’-CGTTGGGATTGGTGACTCTGA

mCol1A1 forward = 5’-GTG TGC GAT GAC GTG CAA TG; reverse = 5’-TTG GGT CCC TCG ACT CCT AC

mFGF23: forward = 5’-GGATCCCCACCTCAGTTCTCA; reverse = 5’-GGAAGTGTCCGGATAGGCTC

mRANKL: forward = 5’-AAA ACG CAG GTT TGC AGG AC; reverse = 5’-GTG AGG TGT GCA AAT GGC TG

mTRAP: forward = 5’-TTC AGG ACG AGA ACG GTG TG; reverse = 5’-CTC TCG TGG TGT TCA GGG TC

Cell proliferation MTS assay : myeloma cells were seeded at 10^4 cells in 100 µL in 96-well plates in RPMI-1640 media with 10% FBS +/- recombinant human FGF23 100 ng/ml, and +/- added calcium chloride (2.5mM and 5mM). Proliferation in response to FGF23 was quantified using the CellTiter 96 aqueous non-radioactive cell proliferation assay (Promega). After incubating for 96 hours, each well was treated with MTS substrate (3-(4,5-dimethylthiazol-2-yl)-5-(3-carboxymethoxyphenyl)-2-(4-sulfophenyl)-2H-tetrazolium) for 1 to 4 hours, after which absorbance at 490nm was recorded using a 96-well plate reader. The quantity of formazan product measured is directly proportional to the number of living cells. Data were graphed as means ± SEM from 3 replicates.

Supplemental References for Methods:

1. Barash U, Cohen-Kaplan V, Arvatz G, Gingis-Velitski S, Levy-Adam F, Nativ O, Shemesh R, Ayalon-Sofer M, Ilan N, Vlodavsky I. A novel human heparanase splice variant, T5, endowed with protumorigenic characteristics. FASEB J. 2010 Apr;24(4):1239-48.
2. Barash U, Arvatz G, Farfara R, Naroditsky I, Doweck I, Feld S, Ben-Izhak O, Ilan N, Nativ O, Vlodavsky I. Clinical significance of heparanase splice variant (t5) in renal cell carcinoma: evaluation by a novel t5-specific monoclonal antibody. PLoS One. 2012;7(12):e51494.
3. Matsumura Y, Aizawa H, Shiraki-Iida T, Nagai R, Kuro-o M, Nabeshima Y. Identification of the human klotho gene and its two transcripts encoding membrane and secreted klotho protein. Biochem Biophys Res Commun 1998;242:p 626-630.

Additional References on Heparanase and EGR1 in Cancer Metastasis and Myeloma:

1. Bolli N, Avet-Loiseau H, Wedge DC, Van Loo P, Alexandrov LB, Martincorena I, Dawson KJ, Iorio F, Nik-Zainal S, Bignell GR, Hinton JW, Li Y, Tubio JM, McLaren S, O' Meara S, Butler AP, Teague JW, Mudie L, Anderson E, Rashid N, Tai YT, Shammas MA, Sperling AS, Fulciniti M, Richardson PG, Parmigiani G, Magrangeas F, Minvielle S, Moreau P, Attal M, Facon T, Futreal PA, Anderson KC, Campbell PJ, Munshi NC. Heterogeneity of genomic evolution and mutational profiles in multiple myeloma. Nat Commun. 2014;5:2997.
2. Barash U, Cohen-Kaplan V, Dowek I, Sanderson RD, Ilan N, Vlodavsky I. Proteoglycans in health and disease: new concepts for heparanase function in tumor progression and metastasis. FEBS J. 2010 Oct;277(19):3890-903.

3. Barash U, Zohar Y, Wildbaum G, Beider K, Nagler A, Karin N, Ilan N, Vlodavsky I. Heparanase enhances myeloma progression via CXCL10 down regulation. Leukemia. 2014 Apr 4. doi: 10.1038/leu.2014.121. [Epub ahead of print]
4. Kelly T, Miao HQ, Yang Y, Navarro E, Kussie P, Huang Y, MacLeod V, Casciano J, Joseph L, Zhan F, Zangari M, Barlogie B, Shaughnessy J, Sanderson RD. High heparanase activity in multiple myeloma is associated with elevated microvessel density. Cancer Res. 2003 Dec 15;63(24):8749-56.
5. Ogishima T, Shiina H, Breault JE, Tabatabai L, Bassett WW, Enokida H, Li LC, Kawakami T, Urakami S, Ribeiro-Filho LA, Terashima M, Fujime M, Igawa M, Dahiya R. Increased heparanase expression is caused by promoter hypomethylation and up-regulation of transcriptional factor early growth response-1 in human prostate cancer. Clin Cancer Res. 2005 Feb 1;11(3):1028-36.
6. Ogishima T, Shiina H, Breault JE, Terashima M, Honda S, Enokida H, Urakami S, Tokizane T, Kawakami T, Ribeiro-Filho LA, Fujime M, Kane CJ, Carroll PR, Igawa M, Dahiya R. Promoter CpG hypomethylation and transcription factor EGR1 hyperactivate heparanase expression in bladder cancer. Oncogene. 2005 Oct 13;24(45):6765-72.
7. Purushothaman A, Uyama T, Kobayashi F, Yamada S, Sugahara K, Rapraeger AC, Sanderson RD. Heparanase-enhanced shedding of syndecan-1 by myeloma cells promotes endothelial invasion and angiogenesis. Blood. 2010 Mar 25;115(12):2449-57.
8. Purushothaman A, Babitz SK, Sanderson RD. Heparanase enhances the insulin receptor signaling pathway to activate extracellular signal-regulated kinase in multiple myeloma. J Biol Chem. 2012 Nov 30;287(49):41288-96.
9. Sanderson RD, Yang Y, Suva LJ, Kelly T. Heparan sulfate proteoglycans and heparanase--partners in osteolytic tumor growth and metastasis. Matrix Biol. 2004 Oct;23(6):341-52.
10. Thompson CA, Purushothaman A, Ramani VC, Vlodavsky I, Sanderson RD. Heparanase regulates secretion, composition, and function of tumor cell-derived exosomes. J Biol Chem. 2013 Apr 5;288(14):10093-9.
11. Yang Y, Macleod V, Bendre M, Huang Y, Theus AM, Miao HQ, Kussie P, Yaccoby S, Epstein J, Suva LJ, Kelly T, Sanderson RD. Heparanase promotes the spontaneous metastasis of myeloma cells to bone. Blood. 2005 Feb 1;105(3):1303-9.
12. Yang Y, Macleod V, Miao HQ, Theus A, Zhan F, Shaughnessy JD Jr, Sawyer J, Li JP, Zcharia E, Vlodavsky I, Sanderson RD. Heparanase enhances syndecan-1 shedding: a novel mechanism for stimulation of tumor growth and metastasis. J Biol Chem. 2007 May 4;282(18):13326-33.
13. Yang Y, MacLeod V, Dai Y, Khotskaya-Sample Y, Shriver Z, Venkataraman G, Sasisekharan R, Naggi A, Torri G, Casu B, Vlodavsky I, Suva LJ, Epstein J, Yaccoby S, Shaughnessy JD Jr, Barlogie B, Sanderson RD. The syndecan-1 heparan sulfate proteoglycan is a viable target for myeloma therapy. Blood. 2007 Sep 15;110(6):2041-8.
14. Zheng L, Pu J, Jiang G, Weng M, He J, Mei H, Hou X, Tong Q. Abnormal expression of early growth response 1 in gastric cancer: association with tumor invasion, metastasis and heparanase transcription. Pathol Int. 2010 Apr;60(4):268-77.

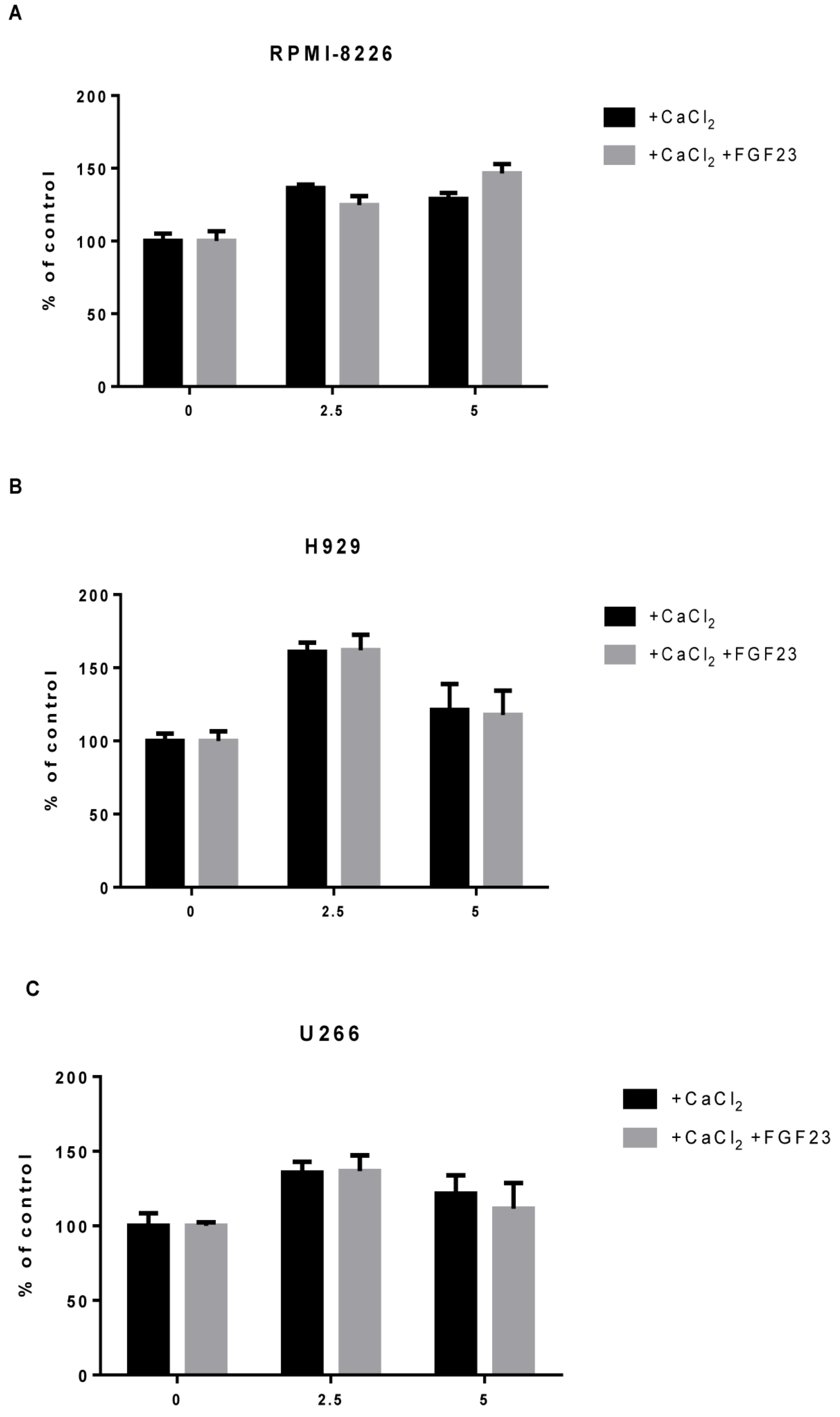

<u>Figure S1:</u> <u>Effects of FGF23 on growth of MM cell lines</u>. MM cells were grown in standard medium (10^5 cells per well in 1 ml) with or without supplemental calcium as indicated and growth over 5 days determined by MTS assay. RPMI-8226 (**A**), H929 (**B**) and U266 (**C**) cells were unaffected by 100ng/mL FGF23, although moderately growth-stimulated by 2.5mM supplemental calcium.

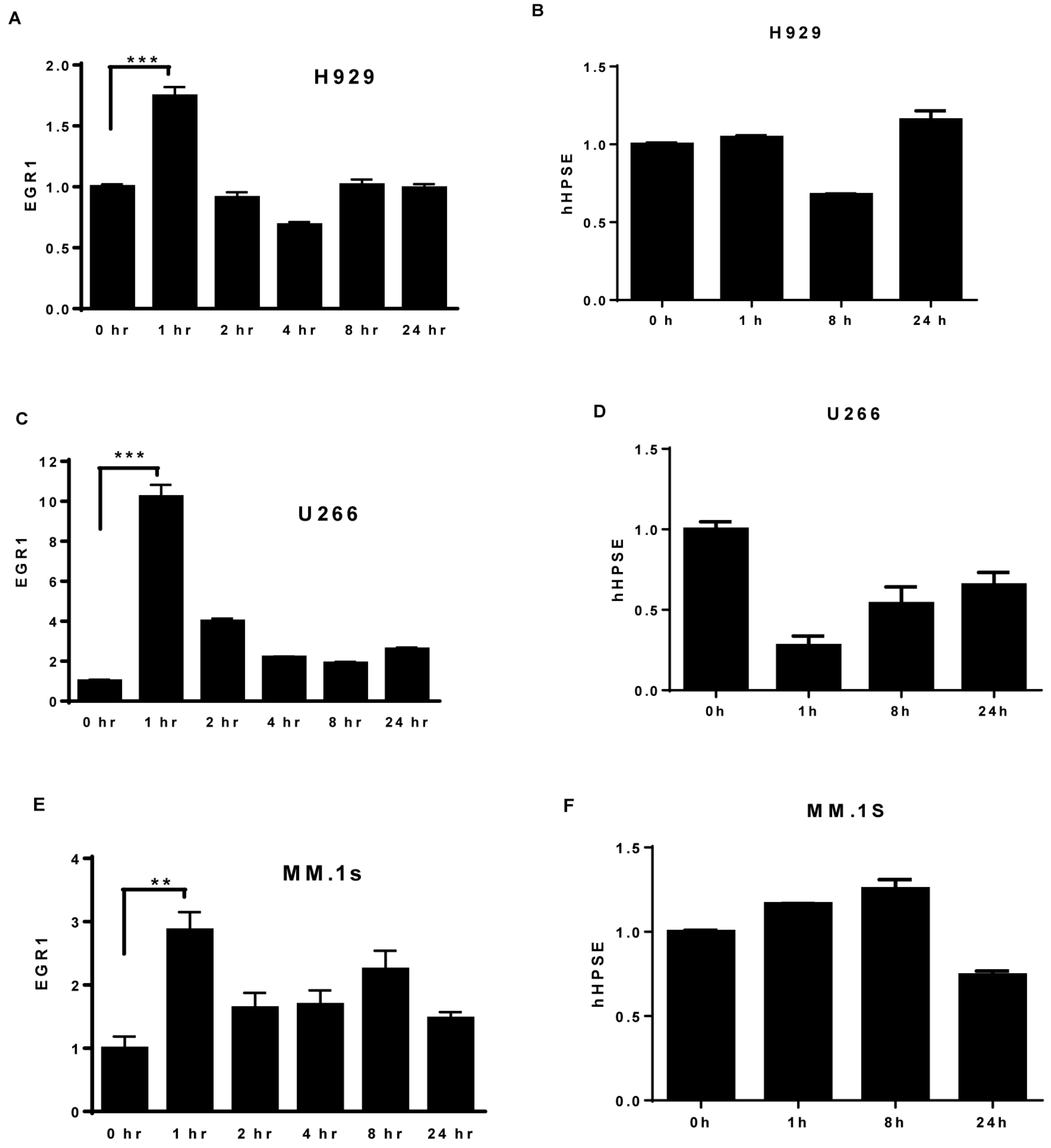

Figure S2: FGF23 regulation of EGR1 and HPSE in MM cell lines. Time courses (0-24hrs) of induction of EGR1 mRNA (left panels) determined by Q-PCR in H929 (**A**), U266 (**C**), and MM.1s (**E**). The increase in EGR1 mRNA (left panels) at one hour was significant versus zero hours at $p < 0.001$ for U266 and H929 and < 0.01 for MM.1s cells. Time courses of induction of full length heparanase (HPSE) mRNA (right panels) for H929 (**B**), U266 (**D**), and MM.1s (**F**), showed that heparanase mRNA was not significantly increased by treatment.

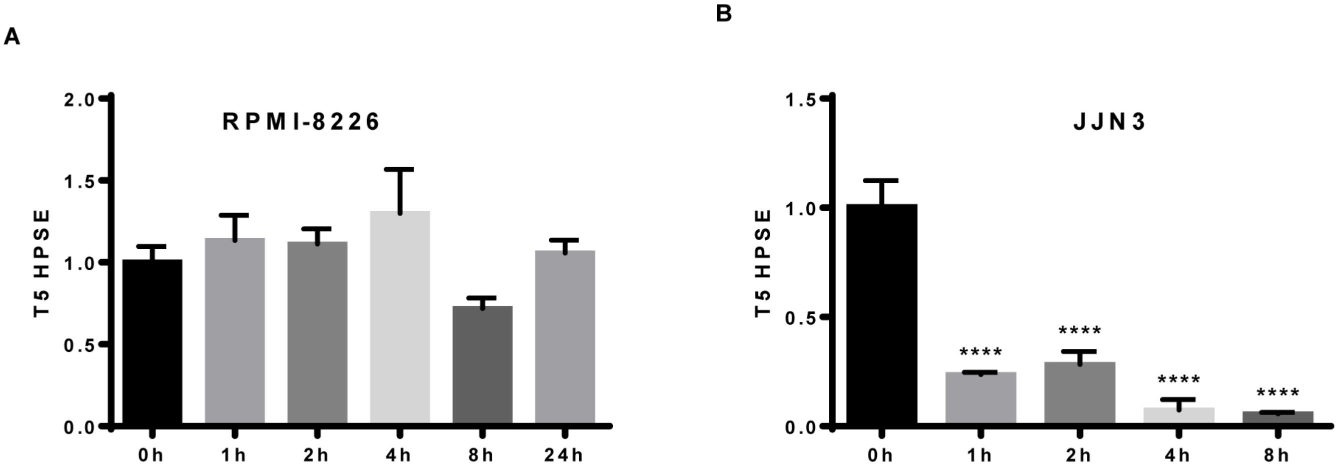

Figure S3: Expression of heparanase T5 isoform. An alternate transcript, T5, of the heparanase gene encodes a short isoform and is highly expressed in renal cancers [1,2], which, like MM when lodged in bone, stimulate osteolytic bone destruction. The T5 isoform was not increased by FGF23 in RPMI-8226 MM cells (**A**) and was decreased in JJN3 cells (**B**). Baseline T5 mRNA concentrations were <10% those for full-length heparanase.

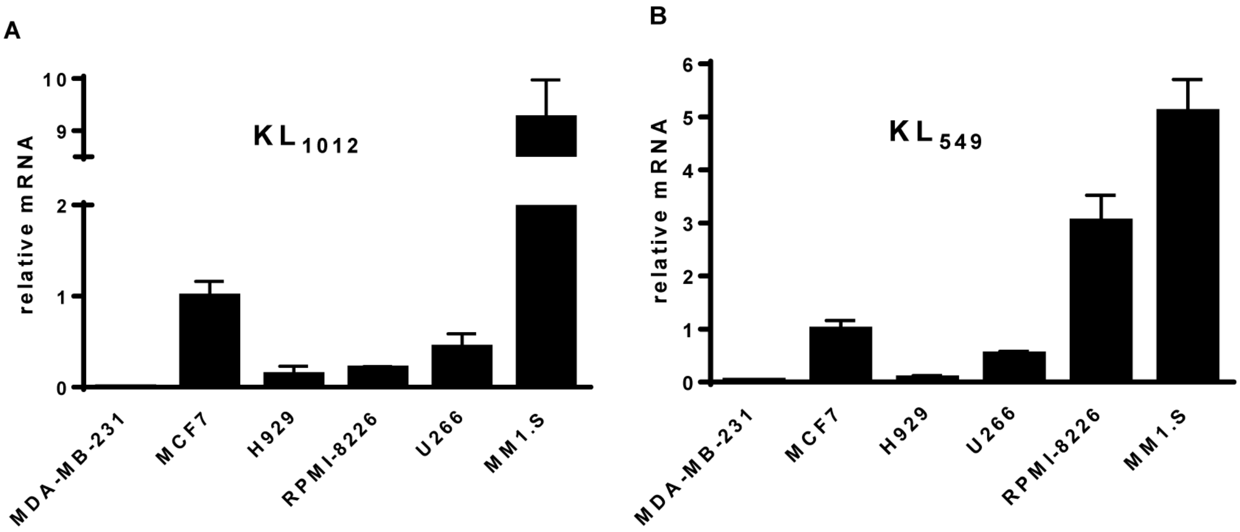

Figure S4: Klotho isoform expression in MM. MM cell lines expressed mRNA encoding the 1012 amino acid membrane precursor for klotho (**A**) and the alternatively spliced mRNA encoding the 549 precursor to the secreted isoform of klotho (**B**) by Q-PCR. MDA-MB-231 and MCF7 are low and high klotho-expressing human breast cancer cell lines used as controls. The two isoforms were distinguished by real time PCR using a common 5' primer and 3' primers specific for the different sequences downstream of the alternatively used splice site [3]. It is likely that the majority of circulating soluble klotho is derived from shedding of an ~980 amino acid form from its transmembrane anchor, rather than from expression of the alternatively spliced transcript, but reagents for evaluating predicted circulating fragments of FGF23 are not available.

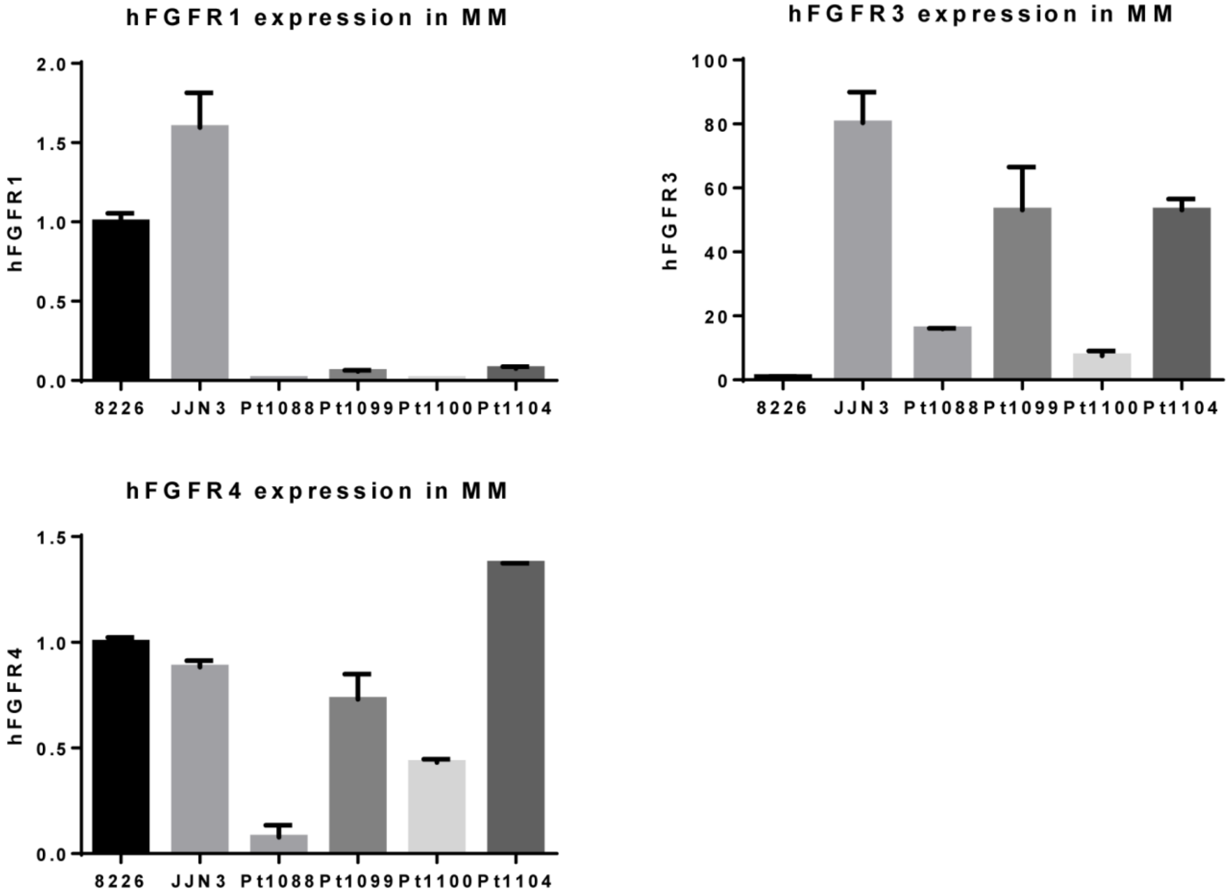

Figure S5: Expression of FGFRs by myeloma cell lines and primary cells. MM patient RNA samples analyzed are the same as those used in main **Figure 1**. PCR primers for FGFRs 1 and 3 recognize RNAs encoding 3c protein isoforms, which are known to form complexes with klotho. In each panel the value for the abundance of the mRNA in RPMI-8226 cells is set as 1. Treatment of 8226 cells for 24hrs with 100ng/ml FGF23 increased all 3 FGFR mRNAs 2-4-fold (not shown).

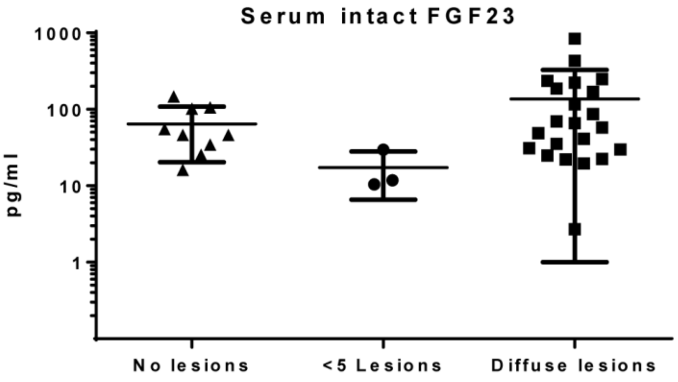

Figure S6: Subgroup analysis of serum intact FGF23 concentrations. Data from **Figure 1A** were analyzed according to characteristics of patient bone lesions. Using skeletal surveys performed at the time of diagnosis, we divided patients into 3 groups based on bone involvement: 1) no lytic lesions (n=9); 2) fewer than 5 lytic lesions (n=3); 3) more than 5 lytic lesions or diffuse skeletal involvement (n=21). The differences in FGF23 among 3 groups were not statistically significant (64.4 ± 44.1, 17 ± 10.7, 136 ± 190 pg/ml, respectively, when the groups were compared using ANOVA on ranks.
